# Notes and News

**Published:** 1891-03-21

**Authors:** 


### Notes and News.

Collected and Communicated.

The thirty-third annual report of the Dental Hospital of London should give satisfaction to all concerned. The institution affords immense benefits upon the poor, no fewer than 48,764 cases having been treated during 1890, being 26,770 in excess of the number in 1874, when the hospital was removed to its present site. The annual subscriptions show a gratifying increase, but there still remains £2,500 of the mortgage debt on the hospital, and the Committee urgently appeal for funds to rid them of this encumbrance incurred by the necessary extension of the hospital.

A painful occurrence is reported from Wheeling, West Virginia. Dr. George Garrison and Dr. George Baird, both prominent physicians, quarrelled over some political question, and although up till then they had been excellent friends, the quarrel became a very bitter one, and the two gentlemen ceased to speak. A short time ago Dr. Garrison, who was City Health Officer, had Dr. Baird arrested for an alleged violation of the health ordinances. This so enraged Dr. Baird that on meeting Dr. Garrison afterwards he threatened him. Since then both men have gone armed, and on the 7th inst. they met, and on Dr. Baird making an insulting remark Dr. Garrison shot him dead with a revolver, and then gave himself up to the police.

A notable feature in the report for 1890 of the Surbiton Cottage Hospital is the evidence of the increasing interest the working classes take in the hospital, the contributions by the friendly societies and clubs showing a considerable increase. On the other hand the subscription list, as compared with that of 1889, exhibits a deficiency of £30 18s. 6d., a condition which causes the Committee much anxiety, as the institution chiefly depends upon this source of income for its maintenance. The number of patients under treatment was practically the same as for the two previous years, but owing to the serious nature of many of the cases the number of days in hospital shows a very considerable increase, attended necessarily with greater expenditure, and there is a deficiency on the year's accounts of £12 19s. 2d.

The Leprosy Commission, consisting of Dr. Buckmaster, Dr. Hautback, and Dr. Rake, which left England last autumn, has now reached Calcutta, after working through the Bombay and Madras Presidencies. The necessity and practicability of segregation, and the great pathological questions of the communicability of leprosy, and heredity, are under close investigation. Many hundreds of lepers have been personally interrogated, and the Commission has received much assistance in this part of the work from the civil surgeons of the districts visited. The Commission's work in India will not be brought to a close before the end of the year, and it is needless to say that the report which embodies the result of so thorough an investigation will be awaited with immense interest.

A prize of the value of £100, open to competitors of all nations, is now being offered by the Royal College of Physicians of Edinburgh, under the Parkin bequest, for the best essay on "The Curative Effects of Carbonic Acid Gas or other forms of Carbon in Cholera, the Different Forms of Fever, and other Diseases."

The 123rd annual report of the Leeds General Infirmary is eminently satisfactory as a record of work done; but in moving its adoption at the meeting of subscribers, Mr. R. B. Jowitt, had to announce that the Board had practically reached the limit of their resources in respect to accommodation, and that the expenditure last year exceeded the income by £764. A new wing is in the course of construction, and will, it is hoped, be ready for occupation next year. An appeal will then be made to the public of Yorkshire for assistance in furnishing the new wing, and for additional annual subscriptions.

The annual meeting of the Hospital Saturday Fund was held on Saturday at the Mansion House, and was presided over by the Lord Mayor. The report stated that the net result of the year's work had enabled the Council not only to rid themselves of a debt of nearly £1,000, carried forward from the previous year's expenditure, but to award to the various hospitals and dispensaries of London £16,000, as compared with £12,000 in 1889, and in addition to carry forward a substantial balance to the credit of this year's account. A resolution was carried to the effect that the meeting heartily approved of the efforts made by the Council to extend the penny-a-week collection among the factories, warehouses, and other places of business throughout the metropolis.

Dr. Littlejohn's report on the prevalence of typhoid in Edinburgh last autumn, discloses some very unfavourable facts about the milk supply, to which the outbreak was attributed. Inquiry having elicited the fact that the seven milk shops in the infected district each received a portion, at least, of their milk, from a particular farm in the country, Dr. Littlejohn visited that farm; there he found that not only was a member of the farm family ill with typhoid, but at the farm itself a state of matters was revealed as highly undesirable in the relation to a milk service as could well be imagined. A badly-drained and filthy byre and scalding house, daily manipulation of milk amid insanitary surroundings, use of polluted water for purposes of can washing, &c., and lastly, want of cleanliness in the family of the dairyman! One only wonders how the milk could have been even apparently fit for consumption.

The Governors of the Bristol General Hospital held their annual meeting at the institution, when the chair was taken by the Mayor. The report, read by the Secretary, showed clearly that the new wards are very much needed, as many as 751 individual cases having been turned away through lack of accommodation. Every bed in the hospital has been constantly occupied for years past. In the out-patient department the work of the hospital has increased nearly 50 per cent. in the last four years. The expenditure for 1890 amounted to £8,994, and as there has been a decrease both in subscriptions and donations, the Committee were obliged to add all legacies received to the annual income, and by this means reduced the balance due to the Treasurer to £782. Mr. Proctor Baker, in seconding the motion of the Mayor that the report be adopted, said that it should be a matter of honour amongst the workmen of Bristol, who received such benefits from the institution, that they should liberally support it. The report was adopted.

MR. BURDETT-COUTTS, M.P., who presided at the fourth annual meeting of the Hospital for Diseases of the Throat, Nose, and Ear, drew attention to the small amount received from subscriptions, and said that he hoped the charitable public would assist the Committee in furnishing the new ward, and increase the income of the hospital by annual subscriptions and donations. The number of attendances of out-patients at this hospital last year was 10,004.

---

ON Tuesday, the 10th inst., in commemoration of the wedding anniversary of H.R.H. the Princess of Wales, the celebrated Danish songstress, Mdlle. Otta Brony, accompanied by Madame Sinico, paid a visit to the British Home for Incurables, at Clapham Rise, and entertained the helpless and suffering inmates with music and singing. The British Home for Incurables was the first charitable institution to receive the honour of the patronage of the Princess of Wales.

---

AT a meeting of the Commissioners of Sewers, on the 10th inst., Mr. Stapley called attention to the alleged insanitary condition of St. Bartholomew's Hospital, and moved that the matter be referred to the Sanitary Committee for consideration and report. Mr. Williamson thought they should have a great deal more information than they possessed before they stigmatised one of their great public institutions as insanitary. Mr. Shaw, as one of the Governors of the Institution, said there was no justification whatever for the motion, as the authorities were doing all that they could in the matter. Dr. Saunders, the City Medical Officer of Health, said that the only notification which had reached him asking for his interference was by an anonymous letter. During the last ten years only six deaths had occurred amongst the hospital employés. The condition of the hospital had been thoroughly sifted by a Committee of the House of Lords, as well as by the Governors, and he should prefer to wait until he had seen Dr. Thomas's report, when he would report to the Commissioners.

---

THE annual meeting of the supporters of the Royal Hospital for Diseases of the Chest, at which the Lord Mayor presided, took place in the board room of the hospital. From a special notice distributed in the room, it appeared that the present financial condition of the hospital is a source of considerable anxiety to the Council. Upwards of £1,000 is owing to the local tradespeople for provisions supplied during October, November, and December last, and at the end of the present month another £1,000 will be owing. The annual subscriptions of the charity amount only to £1,600, whereas the annual expenditure exceeds £6,400; leaving a deficit of nearly £5,000, which has to be found each year, to enable the hospital to carry on its work. The seventy-seventh annual report states that the total number of in-patients treated during the year was 523, whilst the number of out-patients who had availed themselves of the benefits of the institution amounted to no less than 23,666; but although the result of the past year's working is satisfactory, it might have been much more so had the funds permitted the opening of two more wards, and prevented the compulsory refusal of many sad and deserving cases. The sum required to place the finances of the hospital in a satisfactory condition is about £5,000, towards which subscriptions may be sent to the Secretary, Mr. John Harrold, at the hospital, City Road. On the motion of the Lord Mayor the report was adopted. In the course of the speeches which followed, Dr. Gilbert Smith, one of the hon. physicians, stated that in all suitable cases full use had been made of the Koch remedy; the medical treatment of the patients generally being always in accordance with the latest discoveries of science.

THE annual meeting of the Manchester Hospital for Consumption and Diseases of the Throat and Chest was held in the Town Hall, under the presidency of Mr. W. T. Crossley. Mr. C. Behrens, after reading a satisfactory report of the year's work, announced that the Chairman had undertaken to provide a ward containing twelve beds, on condition that the Board of Management undertook the enlargement of the out-patient department. This enlargement has now been commenced. The undertaking will cost about £3,000, towards which sum £2,000 has already been promised. Dr. Ransome, in the course of some observations, said that the City Council was making great efforts to improve the dwellings of the poor, as there could be no doubt that these badly-ventilated back-to-back houses were a great source of disease.

